# Meiosis specific coiled-coil proteins in Shizosaccharomyces pombe

**DOI:** 10.1186/1747-1028-2-14

**Published:** 2007-05-18

**Authors:** Ayami Ohtaka, Takamune T Saito, Daisuke Okuzaki, Hiroshi Nojima

**Affiliations:** 1 Department of Molecular Genetics, Research Institute for Microbial Diseases, Osaka University, 3-1 Yamadaoka, Suita, Osaka 565-0871, Japan; 2 Department of Genetics, Harvard Medical School 77 Avenue Louis Pasteur, New Research Building, Room 334, Boston, MA 02115, USA

## Abstract

Many meiosis-specific proteins in *Schizosaccharomyces pombe *contain coiled-coil motifs which play essential roles for meiotic progression. For example, the coiled-coil motifs present in Meu13 and Mcp7 are required for their function as a putative recombinase cofactor complex during meiotic recombination. Mcp6/Hrs1 and Mcp5/Num1 control horsetail chromosome movement by astral microtubule organization and anchoring dynein respectively. Dhc1 and Ssm4 are also required for horsetail chromosome movement. It is clear from these examples that the coiled-coil motif in these proteins plays an important role during the progression of cells through meiosis. However, there are still many unanswered questions on how these proteins operate. In this paper, we briefly review recent studies on the meiotic coiled-coil proteins in *Sz. pombe*.

## Background

Meiosis, a fundamental biological phenomenon conserved from yeast to mammals, is a specialized form of cell division that generates haploid gametes from diploid parental cells and that presumably evolved from mitosis. The fission yeast, *Schizosaccharomyces pombe*, is an ideal model organism to study the molecular mechanism of meiotic regulation because meiosis can be induced from a mitotic cell simply by removing the nitrogen source from the growth medium. *Sz. pombe *is most stable in the haploid state and behaves asexually in rich nutritional environments. Upon nitrogen deprivation, two haploid cells with opposite mating types conjugate without delay, after which their nuclei fuse and undergo meiosis. After a single round of DNA replication, the nucleus (or chromosome) begins to oscillate between the cell poles and acquires an elongated shape called a horsetail. During this horsetail movement, pairing of homologous chromosome and genetic recombination occur, which generates new allelic combinations in the resulting gametes, thereby increasing the genetic diversity of the offspring. Subsequently, two rounds of successive chromosome segregation, called meiosis I (MI) and meiosis II (MII), occur, which results in a 2-fold reduction in chromosome number. During a process called sporulation, intracellular formation of a double layer forespore membrane (FSM) occurs, which matures into spore walls. Thus, one cell produces one ascus containing four spores.

The expression of specific genes that regulate meiosis are induced throughout the process. These meiotic genes are classified as early, middle and late genes based on the timing of their expression during meiosis. These genes are further classified according to their structural motifs. One structural motif that is frequently observed is the coiled-coil motif, a ubiquitous protein folding structure containing seven-residue repeats which form alpha-helices that wrap around each other [1-4]. Understanding the function of meiotic coiled-coil proteins is important not only to understand their direct role in meiotic regulation but also their indirect role through their interaction and regulation of other meiotic coiled-coil proteins. Indeed, some of the meiotic coiled-coil protein interactions in *Sz. pombe *are pivotal during meiosis. Here, we describe recent findings on the meiotic coiled-coil proteins of *Sz. pombe*.

### Meiosis specific coiled-coil proteins of *Sz. pombe*

**Figure 1:**
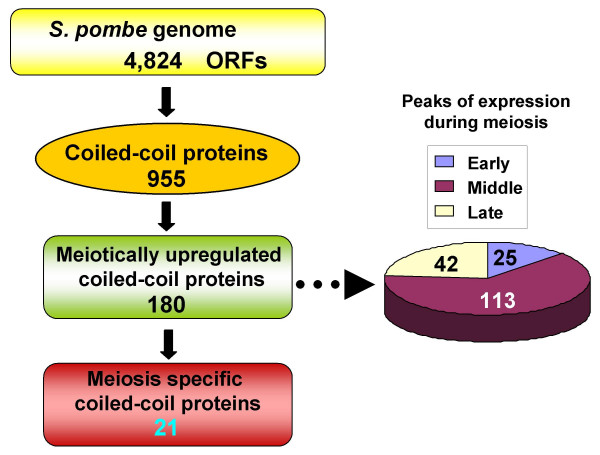
The numbers of open reading frames (ORFs), coiled-coil proteins, meiotically upregulated coiled-coil proteins, and meiosis specific coiled-coil proteins that are predicted from the *Sz. pombe *genome. The 180 genes that encode meiotically upregulated coiled-coil proteins are divided into three categories according to their peak in expression levels (early, middle and late).

The genome sequence of *Sz. pombe *[5] has 4,824 open reading frames (ORFs) (which can be viewed through the *Sz. pombe *GeneDB [6]) which encode 955 proteins containing coiled-coil motifs (Fig. 1). The genome-wide transcriptome analysis [7] identified 180 genes which encoded proteins with coiled-coil motifs whose expressions are upregulated during meiosis. Among those 180 genes, 25 (13.9%), 113 (62.8%) or 42 (23.3%) are early, middle or late genes, respectively. Expression analysis for each gene has shown that at least 21 of these genes are expressed solely during meiosis and not during mitosis [8-19]. These 21 meiosis specific coiled-coil proteins include 8, 11 or 2 early, middle or late genes, respectively (Table 1). In contrast, 159 out of 180 genes are also expressed during mitosis although their expression levels are low compared with their expression during meiosis. It remains to be determined if there are other genes whose expression is specifically upregulated during meiosis.

**Table 1:** List of meiosis-specific coiled-coil proteins.

Gene Name	ORF Name	Amino acids	Category	Protein Features
*tht1*	SPAC13C5.03	543	early	nuclear membrane protein involved in karyogamy
*meu13*	SPAC222.15	216	early	meiotic expression upregulated
*mcp7*	SPAC13A11.03	217	early	meiosis specific coiled-coil protein
*mcp6/hrs1*	SPBC582.06C	327	early	meiosis specific coiled-coil protein
*mcp5/num1*	SPBC216.02	968	early	meiosis specific coiled-coil protein
*dhc1*	SPAC1093.06C	4196	early	dynein heavy chain
*ssm4*	SPAC27D7.13C	670	early	p150-Glued
*eta2*	SPAC31G5.10	569	early	Myb family
*sgo1*	SPBP35G2.03C	319	middle	shugoshin
*meu14*	SPBC1347.03	335	middle	meiotic expression upregulated
*mcp4*	SPBC16E9.08	355	middle	meiosis specific coiled-coil protein
*mcp3*	SPAC1006.04C	952	middle	meiosis specific coiled-coil protein
*meu1*	SPAC1556.06	776	middle	meiotic expression upregulated
*meu18*	SPBC409.11	553	middle	NLS
*meu23*	SPCC613.11C	254	middle	B13958 domain
*spn5/mde9/meu28*	SPAC24C9.15C	464	middle	septin
*mde4*	SPBC6B1.04	421	middle	mei4 dependent protein
*mcp2*	SPCC1682.08C	703	late	RNA-binding protein
*mcp1*	SPAC1687.10	661	late	meiosis specific coiled-coil protein
*meu6*	SPBC428.07	651	late	lysine-rich protein
*mpf1*	SPAC4G9.05	581	late	meiotic PUF family protein 1

**Figure 2:**
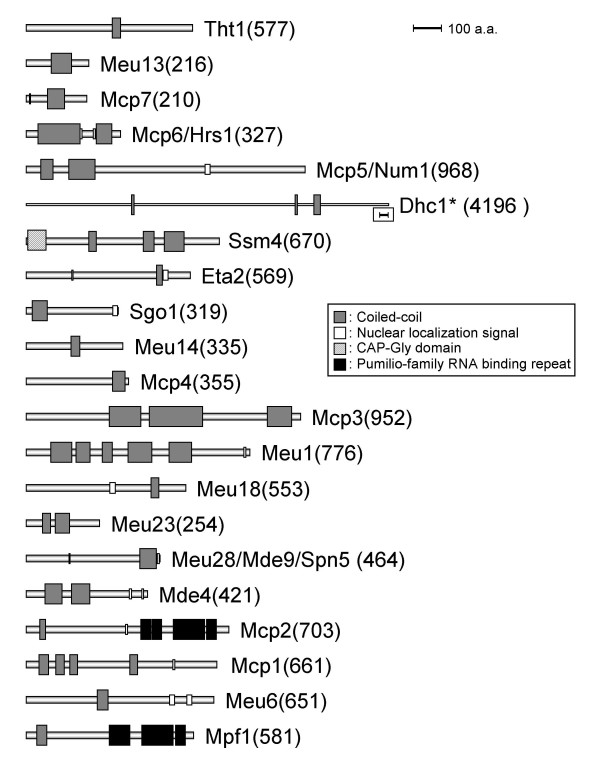
The position, number and size of the coiled-coil motifs in each protein are schematically depicted by grey boxes. Additional motifs including the NLS (white box), microtubule-binding CAP-Gly domain (shaded box) and Pumilio-family RNA binding repeat (black box) are also shown. The number following each protein name represents the size of amino acids of each protein. The size of Dhc1 alone is reduced to 30% of its original size. Bars, 100 amino acids.

The size, number and position of the coiled-coil motifs from meiosis specific coiled-coil proteins so far reported are schematically presented in Figure 2. Since coiled-coil motifs consist of two or more alpha-helices that wind together in a supercoil, the motif can vary in size depending on the number of alpha-helices that form versatile folds in each protein. Moreover, the locations and number of coiled-coil motifs in a protein impacts other interacting proteins. Indeed, 9 out of 21 meiosis specific coiled-coil proteins (Mcp1, Mcp3, Mcp5, Mcp6, Meu1, Meu23, Mde4, Ssm4, Dhc1) have more than two coiled-coil motifs. In particular, the coiled-coil motif region occupies a large portion of Mcp3, Mcp6, Meu1 and Meu23 suggesting the indispensable roles of coiled-coil motifs for the function of these proteins. The other proteins carry only a single and comparatively short stretch of coiled-coil sequence. However, this may not necessarily suggest a less important role for these coiled-coil folds in protein function.

### Putative functions of early coiled-coil genes

**Figure 3:**
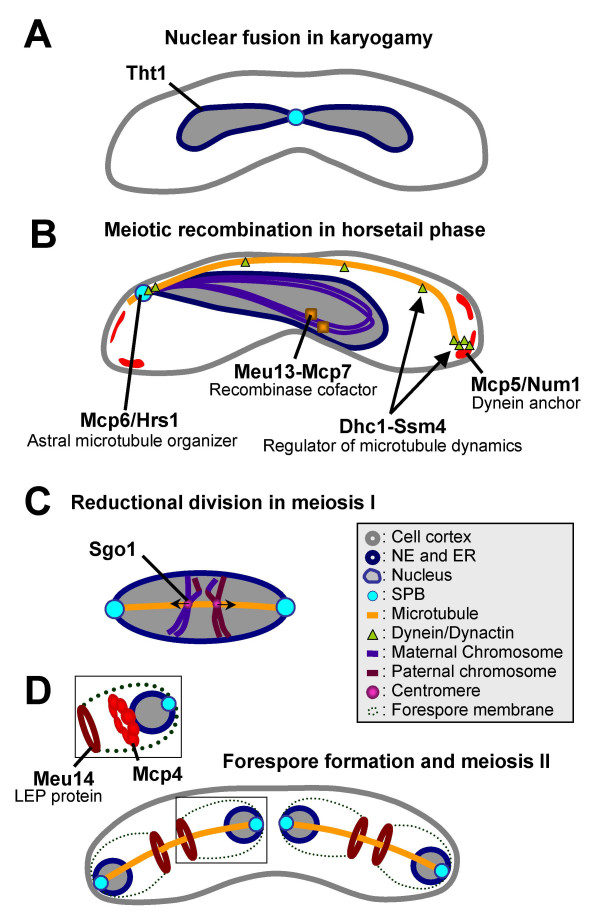
Schematic representation of the typical subcellular localization of meiosis specific coiled-coil proteins. (A) Tht1 is detected at the nuclear envelope (NE) and endoplasmic reticulum (ER) during karyogamy. (B) Mcp6/Hrs1, Meu13-Mcp7 and Mcp5/Num1 localize to SPB, chromatin and cell cortex, respectively. Dhc1 and Ssm4 localize to SPB microtubule and cell cortex. (C) Sgo1 localizes to centromeres and protects the centromeric cohesin from degradation during anaphase I. (D) Meu14 forms a ring-shaped structure at the aperture of fore spore membrane (FSM) during the sporulation process. Mcp4 also forms a ring and is localized between Meu14 and nucleus.

The physiological role of the eight early meiosis specific genes that encode coiled-coil proteins have been analyzed in detail. Subcellular localizations and functions of these proteins during meiosis are diverse. For example, Tht1, a type I membrane glycoprotein with a single coiled-coil motif in the middle of the molecule, plays a key role in karyogamy, the nuclear fusion process occurring at the start of meiosis in which two haploid nuclei fuse to produce a diploid nucleus [9]. Tht1 is present in the nuclear envelope and endoplasmic reticulum (ER) during karyogamy (Fig. 3A), but is not observed during later stages of meiosis. In the *tht1 *mutant, nuclear congression and spindle pole body association occurs but fusion of nuclear envelopes is blocked. Nonetheless, meiosis continues which results in the generation of aberrant asci. The role of the coiled-coil domain in Tht1 during nuclear envelope fusion remains elusive.

Meu13, another meiosis specific coiled-coil protein, is required for proper homologous pairing and recombination [13]. It is present on meiotic chromatin and plays a pivotal role in chromosome pairing through a recombination-independent mechanism, promoting proper homologous pairing and recombination. Mcp7, another meiosis specific coiled-coil protein, forms a complex with Meu13 during meiosis [16]. Since a single coiled-coil motif occupies the central part of each protein, it is likely that their interaction is mediated by coiled-coil motif. Both *meu13*Δ cells and *mcp7*Δ cells have reduced recombination rates and spore viability and generate spores with abnormal morphology. The coiled-coil motifs in the mouse homologs Hop2 (Meu13) and Mnd1 (Mcp7) are essential for their interaction [20]. The Hop2-Mnd1 complex ensures that homologous chromosomes properly synapse during meiosis by acting in concert with Rad51 and Dmc1 to promote the strand invasion step (D-loop formation) of homologous recombination [20].

Mcp6/Hrs1 is required for proper astral microtubule positioning to maintain the horsetail movement of chromosomes [17,21]. Mcp6/Hrs1 is present at the start of karyogamy where it localizes to the spindle-polebody (SPB) before it is degraded prior to chromosome segregation at meiosis I. Although the allelic recombination rates of the *mcp6*Δ cells are 10–40% lower than of the wild-type, the ectopic recombination rates are doubled. This is likely due to abnormal homologous pairing resulting from aberrant horsetail movement in this mutant [17]. Hrs1 directly interact with Alp4, a component of gamma tubulin ring complex (γ-TuRC), and Mto1, a gamma tubulin recruiting protein [21]. In *hrs1*Δ cells, Alp4 becomes dispersed around nucleus from its original localization at SPB in early meiosis I. On the other hand, overexpression of Hrs1 at mitotic interphase causes the homogeneously distributed Alp4 to accumulate to SPB and induces movement of chromosome and formation of aster. From these results, it is proposed that Hrs1 recruits Alp4 to SPB, promotes the formation of horse tail astral array (HAA) after formation of pre-HAA by Mto1, attracts minus-end microtubule to SPB via γ-TuRC and stabilizes it [21]. It is notable that all of the interacting proteins with Hrs1 (full length) so far identified, such as Alp4, Mto1 and Kms1, posses coiled-coil motif, although their physiological significance remains elusive.

Mcp5/Num1 is also required for horsetail chromosome movement [18,22]. Although in budding yeast the dynein anchor Num1, a homolog of Mcp5, is expressed and functions during both meiosis and mitosis, Mcp5 in *Sz. pombe *is expressed and functions only during meiosis. Mcp5/Num1 is located at the cell cortex and plays a role in anchoring the cortical dynein in meiotic cells [18,22]. The repeat unit just after the coiled-coil motifs is essential for the complete function of Mcp5/Num1 because mutants which lack the repeat unit in Num1/Mcp5 display aberrant chromosome oscillation probably due to failure in interaction with dynein [22]. This defect results in a decline in recombination rate and in abnormal spore formation [18]. However, physiological roles of coiled-coil motifs remain elusive. Function of Mcp5 during meiosis is summarized as follows. Namely, when the SPB (Fig. 3B, blue circle) and the horsetail chromosome reach the cell cortex, the microtubules carrying the SPB and chromosome extend toward the opposite end. The dynein complex (Fig. 3B, green triangle) on the end of these microtubules is then trapped and anchored by Mcp5 (red shapes). This may stimulate minus-end motor activity which leads to the cortical sliding of the microtubules. Subsequently, the nucleus turns and is pulled towards the opposite side of the cell. When SPB arrives at the cell cortex where dynein is anchored by Mcp5, the pulling force is gradually weakened and the microtubules behind the horsetail chromosome will extend to the opposite end of the cell.

Cytoplasmic dynein is a large multisubunit complex composed of two motor proteins, the dynein heavy chains (DHC), and several associated subunits that have been named light, light intermediate, and intermediate chains [23]. The dynein motor complex transports various cellular cargos by walking along microtubules from its plus-end to minus-end. Unlike mammalian cells, the DHC subunit of *Sz. pombe*, Dhc1, that carries an ATP-dependent minus-end directed motor activity, is expressed only during meiosis and localizes at astral microtubules and the SPB at meiotic prophase I [10,24]. Dhc1 plays a pivotal role in horsetail chromosome movement and homologous chromosome pairing by walking to the minus-end of microtubules when it is anchored to the cell cortex by dynein anchor protein [10,25]. Recent observation indicates that the formation of bouquet arrangement of chromosomes at the telomeres, or telomere clustering, plays an essential role in homologous chromosome pairing and that Bqt1 and Bqt2 connect telomeres to the SPB by forming a bridge between Rap1 (a telomere protein) and Sad1 (an SPB protein) [26]. Considering that dynein light chain (dlc1), a component of dynein complex, interacts with Kms1 (an SPB protein) [27] and that the Sad1 body (Sad1 outside SPB) on nuclear membrane remains dispersed in a *kms1*Δ mutant [28], it is interesting to explore if the dynein motor activity is required for telomere clustering as a driving force [26].

The *ssm4*^+ ^gene, a high copy-number suppressor gene of meiotic arrest encodes a meiosis specific coiled-coil protein carrying a microtubule-binding motif at its N-terminus [8]. Ssm4 (p150-Glued protein) is a component of dynactin, a large multi-subunit complex implicated in cytoplasmic dynein-driven motility, which interacts directly with the cytoplasmic dynein complex and mediates the binding of dynein to cargo such as membranes [29]. Expression of Ssm4 is induced in response to mating pheromone at the early stage of meiosis, when it is required for the horsetail movement of chromosome [24]. Like Dhc1, Ssm4 localizes at SPB and microtubule of horsetail phase. Loss of Ssm4 causes abnormal chromosome movement due to failure in dynein anchoring at the junction of microtubule and cell cortex, which results in frequent generation of asci containing less than four spores [24]. Although the direct evidence is lacking, the coiled-coil motif in Ssm4 may be important for its full function, because the coiled-coil motif of rat p150-Glued protein plays a key role in its direct binding to the dynein intermediate chain [30].

Eta2, also called Plx1 after *multiplex transcript *[31], is a putative transcriptional factor homologous to the mammalian oncogene Myb. This region of the genome, where Plx1 mRNA is transcribed, generates variable sizes of transcripts in a meiosis specific manner [31]. Understanding its physiological role requires further analysis.

### Putative functions of middle coiled-coil genes

Studies of the physiological roles of the eleven middle meiotic coiled-coil genes have revealed their pivotal roles in proper meiotic progression, although the significance of the coiled-coil motif for their functions remains elusive. For example, Sgo1 (shugoshin) cooperates with protein phosphatase 2A (Par1) to protect Rec8-containing centromeric cohesin from degradation during meiosis I [15,32-35]. Sgo1 is also a component of the spindle checkpoint which monitors chromosome alignment on the mitotic spindle, and is required for sensing tension between sister chromatids during mitosis [36]. Thus, Sgo1 provides a molecular link between sister chromatid cohesion and tension-sensing at the kinetochore-microtubule interface and activates the mitotic spindle checkpoint when spindle tension is perturbed [37]. Both Asn-29 and Ile-50 in the coiled-coil domain are required for full function of Sgo1 [15]. Fission yeast have a second Shugoshin family member, Sgo2, which is not meiosis specific and controls docking of the passenger proteins on centromeres upon checkpoint activation in early mitotic cells [39,40]. Sgo2 promotes the localization of Aurora kinase complex to the pericentromeric region *via *interaction with Bir1 (Survivin) component, and plays a role in correction of erroneous attachment of kinetochores, thereby enabling tension-generating attachment.

Meu14 is dynamic during meiosis [14]. It is first observed inside the nuclear region at prophase II, after which it accumulates beside the two SPBs at metaphase II. Thereafter, it forms two ring-shaped structures that surrounded the nucleus at early anaphase I. At this stage, Meu14 appears to localize at the border of the forespore membrane that later develops into spore walls at the end of sporulation. It is then degraded at post-anaphase II. Meiotic *meu14*Δ cells are highly tetranucleate, form aberrant forespore membranes, and fail to produce asci. Thus, Meu14 participates in both nuclear division during meiosis II and in formation of the forespore membrane.

Mcp4 is required for the correct positioning of F-actin during meiosis [19]. Expression of Mcp4 is induced at the horsetail phase and continues to be expressed through sporulation. The subcellular localization of Mcp4 is dynamic during meiosis, being located along side F-actin. At metaphase II, Mcp4 assembles at the lagging face of the dividing nuclei, being sandwiched between F-actin and the nucleus. The shape and viability of *mcp4*Δ spores are normal, but they are sensitive to NaCl. F-actin, which is normally dumbbell-shaped at anaphase II, adopts an abnormal balloon-shape in *mcp4*Δ cells. The coiled-coil motif at the C-terminus of Mcp4 appears to be essential for its full function because ectopically expressed Mcp4 lacking the C-terminal domain is aberrantly localized, losing its proper sandwiched distribution between F-actin and the nucleus.

Mcp3 is also a meiosis-specific protein with three coiled-coil domains, whose expression profile and subcellular localization during meiosis is similar to that of Mcp4 [19] (Ohtaka et al., unpublished data; see Additional file 1). Mcp3 is punctuate throughout the cytoplasm in Metaphase I and perinuclear during anaphase I. It then clumps into four aggregates in anaphase II, before being localized to the inner spore periphery at the end of meiosis (see Additional file 2). Subcellular distribution of Mcp3-GFP is distinct from those of Golgi/endosome structures. Mcp3 and Mcp4 only partially co-localize and appear to be tightly juxtaposed. Although the autolysis of the mother cells to four spored asci was remarkably delayed, the morphology and viability of *mcp3*Δ spores are normal (see Additional file 3).

The expression of the middle genes Meu1, Meu18, Meu23 and Meu28/Mde9/Spn5 (a meiotic septin) are abruptly induced during meiosis, but their function remains unknown [12]. Meu1 mRNA is one of a dozen meiosis-specific transcripts that is selectively removed by binding to the cis-acting motif of Mmi1 if it is expressed during vegetative growth [41]. Meu1 has five coiled-coil motifs in the middle of the molecule and a nuclear localization sequence (NLS) at its C-terminus, suggestive of a nuclear function. However, disruption of *meu1*^+ ^gene results in no apparent phenotype (Shigehisa et al., unpublished observation). Expressions of Meu1, Meu18, Meu28 and Mde4 are regulated by Mei4, a meiosis specific transcription factor [42]. Mcp1 and Mcp2 are meiosis-specific proteins with four and one coiled-coil domains, respectively, and whose expressions are induced at the sporulation phase of meiosis. Nonetheless, disruption of *mcp1*^+ ^and *mcp2*^+ ^genes causes no sporulation defect (our unpublished observation).

### Putative functions of late genes

The function of the two late genes is also unclear. Meu6 was identified as a gene whose expression is only upregulated during meiosis [12]. Meu6 harbors one coiled-coil motif and an NLS, which suggests that it has a nuclear role. Mpf1 was identified through a comprehensive screening of meiosis specific RNA binding proteins. It contains a RNA binding motif that belongs to the PUF family and a coiled-coil motif at its N-terminus [43].

During the late phase of meiosis in *Sz. pombe*, several coiled-coil proteins are recruited to the SPBs and play indispensable roles in FSM assembly [44], although the expression of these proteins are not necessarily specific to meiosis. For example, Spo15, a component of the spindle pole body containing a coiled-coil motif, plays an essential role in the initiation of spore membrane formation, although its expression is detected in both meiotic and vegetative cells [45]. Sec9, a component of the SNARE (soluble N-ethyl-maleimide-sensitive factor attachment protein receptor) complex, is required for both vegetative growth and normal spore formation [46]. Expression of the *sec9*^+ ^gene is strongly induced during sporulation in a Mei4 dependent manner, although *sec9*^+ ^mRNA is also detected in vegetative cells. In sec9 mutant cells, the FSM was properly initiated near spindle pole bodies during meiosis II, but the subsequent membrane extension was severely impaired.

## Conclusion and perspectives

As described above, coiled-coil motifs play pivotal roles for the proper function of the Meu13-Mcp7 complex. Meiosis specific coiled-coil proteins, Meu13 and Mcp7, form a complex probably through an interaction with their coiled-coil motifs and play a pivotal role in meiotic recombination. Mcp6/Hrs1 is required for proper astral microtubule positioning to maintain the horsetail movement of chromosomes, and all of its interacting proteins possess coiled-coil motifs. In addition, Mcp5/Num1 regulates horsetail chromosome movement by anchoring dynein, and the repeat unit just after the coiled-coil motifs is essential for its complete function. Meu14 regulates FSM formation, and a single coiled-coil motif in the central region of the molecule appears to be important for its function. Although the significance of the coiled-coil domain in these proteins is partly known, the roles of coiled-coil motifs for other meiosis specific proteins remain unclear. More experiments are required to understand their roles during the progression through meiosis.

## Competing interests

The author(s) declare that they have no competing interests.

## Authors' contributions

A.O., T.T.S., D.O. and H.N. drafted the manuscript and designed the figures and table. All of the authors have read and approved the final manuscript.

## Supplementary Material

Additional File 1 Expression profiles of Mcp3 during meiosis of *Sz. pombe*. (A) Meiotic expression of *mcp3*^+ ^as assessed by Northern blot analysis. *h*^+^*/h*^-^(CD16-1) and *h*^-^*/h*^- ^(CD16-5) diploid cells were subjected to nitrogen starvation, which induces CD16-1 but not CD16-5 to enter meiosis (upper panels). The cells were collected at 2 hour intervals and the total RNAs were blotted and probed with the *mcp3*^+ ^ORF, respectively (lower panels). The RNAs were also probed with the *aro3*^+ ^ORF as a loading control. (B) Meiotic expression of Mcp3-9myc protein as assessed by Western blot analysis. The *h*^-^*/h*^-^*pat1-114 mcp4*^+^-3ha *mcp3*^+^-9myc strain was induced to enter meiosis synchronously by a temperature shift and the cells were collected at 30 min intervals (upper panels) for protein extraction, blotting and probing with anti-Myc antibody (lower panels). Meu13 expression was also analyzed by using an anti-Meu13 antibody to help identify the meiotic stage at each timepoint. The tubulin levels were also examined as a loading control. At each timepoint in (A) and (B), the frequency of cells with one, two, three or four nuclei was determined by counting at least Hoechst 33342-stained 200 cells under a microscope. Thus, the upper panels show the stage of meiosis. Click here for file

Additional File 2 Subcellular localization of Mcp3 during meiosis and sporulation. (A) A homothallic haploid cell *h*^90^*mcp3*^+^-gfp was cultured in EMM with appropriate supplements and transferred to EMM-N to induce meiosis. Cells in different stages of meiosis were stained with Hoechst 33342 to label DNA (blue) and Mcp3-GFP (green) was monitored under a fluorescence microscope. The merge of the two channels are shown in the rightmost panels. Bar, 10 μm. (B) FM4-64 uptake in living *mcp3*^+^-gfp meiotic cells. Cells were cultured in EMM medium containing appropriate supplements and transferred to EMM-N medium to induce meiosis. Meiotic cells producing Mcp3-GFP (green) were incubated with the fluorescent dye FM4-64 (red) to visualize Golgi/endosomes. It appears that Mcp3-GFP is distinct from the Golgi/endosome structures. Bar, 10 μm. (C) Mcp3 associates transiently with Mcp4 during meiosis. A homothallic haploid strain bearing *mcp3*^+^-9myc *mcp4*^+-^3ha was induced to enter meiosis and then fixed chemically. Cells were stained with Hoechst 33342, anti-myc, and anti-HA antibodies to visualize the DNA (blue), Mcp3-9myc (green), and Mcp4-3HA (red) by fluorescence microscopy, respectively. Bar, 10 μm. Click here for file

Additional File 3 Autolysis of *mcp3*Δ asci is remarkably delayed compared to wild type asci.Description: Wild type (*mcp3*^+^/*mcp3*^+^) asci autolyse 48 hrs after meiotic induction on malt extract (ME) plates(left panel), while almost no *mcp3*Δ/*mcp3*Δ asci are autolysed by this time (right panel). It is only 68 hrs after meiotic induction that most of the *mcp3*Δ/*mcp3*Δ asci are liberated from asci (data not shown). The *mcp3*^+^/*mcp3*Δ strain shows the intermediate phenotype (middle panel). Click here for file
