# Supplementary figures and images for: Meiosis specific coiled-coil proteins in Shizosaccharomyces pombe
Source: Cell Div. 2007 May 18;2:14. doi: 10.1186/1747-1028-2-14 (PMC1885245; doi:10.1186/1747-1028-2-14)

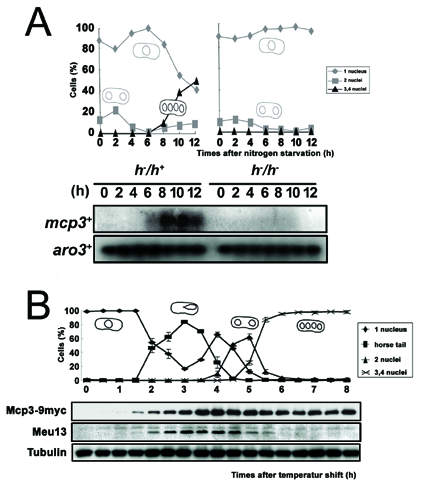

Supplement: Additional File 1 — Expression profiles of Mcp3 during meiosis of Sz. pombe. (A) Meiotic expression of mcp3+ as assessed by Northern blot analysis. h+/h-(CD16-1) and h-/h- (CD16-5) diploid cells were subjected to nitrogen starvation, which induces CD16-1 but not CD16-5 to enter meiosis (upper panels). The cells were collected at 2 hour intervals and the total RNAs were blotted and probed with the mcp3+ ORF, respectively (lower panels). The RNAs were also probed with the aro3+ ORF as a loading control. (B) Meiotic expression of Mcp3-9myc protein as assessed by Western blot analysis. The h-/h-pat1-114 mcp4+-3ha mcp3+-9myc strain was induced to enter meiosis synchronously by a temperature shift and the cells were collected at 30 min intervals (upper panels) for protein extraction, blotting and probing with anti-Myc antibody (lower panels). Meu13 expression was also analyzed by using an anti-Meu13 antibody to help identify the meiotic stage at each timepoint. The tubulin levels were also examined as a loading control. At each timepoint in (A) and (B), the frequency of cells with one, two, three or four nuclei was determined by counting at least Hoechst 33342-stained 200 cells under a microscope. Thus, the upper panels show the stage of meiosis. [file 1747-1028-2-14-S1.tiff]

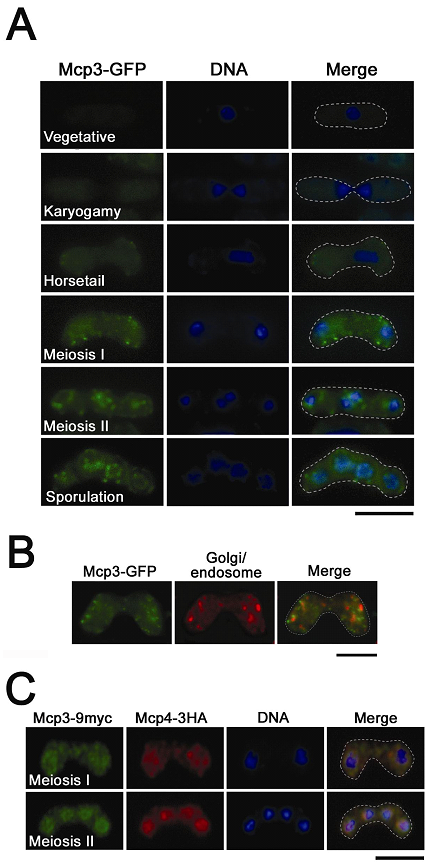

Supplement: Additional File 2 — Subcellular localization of Mcp3 during meiosis and sporulation. (A) A homothallic haploid cell h90mcp3+-gfp was cultured in EMM with appropriate supplements and transferred to EMM-N to induce meiosis. Cells in different stages of meiosis were stained with Hoechst 33342 to label DNA (blue) and Mcp3-GFP (green) was monitored under a fluorescence microscope. The merge of the two channels are shown in the rightmost panels. Bar, 10 μm. (B) FM4-64 uptake in living mcp3+-gfp meiotic cells. Cells were cultured in EMM medium containing appropriate supplements and transferred to EMM-N medium to induce meiosis. Meiotic cells producing Mcp3-GFP (green) were incubated with the fluorescent dye FM4-64 (red) to visualize Golgi/endosomes. It appears that Mcp3-GFP is distinct from the Golgi/endosome structures. Bar, 10 μm. (C) Mcp3 associates transiently with Mcp4 during meiosis. A homothallic haploid strain bearing mcp3+-9myc mcp4+-3ha was induced to enter meiosis and then fixed chemically. Cells were stained with Hoechst 33342, anti-myc, and anti-HA antibodies to visualize the DNA (blue), Mcp3-9myc (green), and Mcp4-3HA (red) by fluorescence microscopy, respectively. Bar, 10 μm. [file 1747-1028-2-14-S2.tiff]

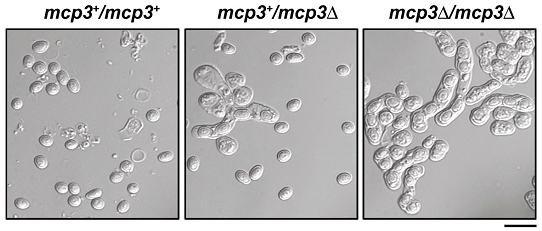

Supplement: Additional File 3 — Autolysis of mcp3Δ asci is remarkably delayed compared to wild type asci.Description: Wild type (mcp3+/mcp3+) asci autolyse 48 hrs after meiotic induction on malt extract (ME) plates(left panel), while almost no mcp3Δ/mcp3Δ asci are autolysed by this time (right panel). It is only 68 hrs after meiotic induction that most of the mcp3Δ/mcp3Δ asci are liberated from asci (data not shown). The mcp3+/mcp3Δ strain shows the intermediate phenotype (middle panel). [file 1747-1028-2-14-S3.tiff]
